# The motivational mechanism behind college students’ willingness to participate in rural revitalization--a case study of the “Furong Scholars •Rural Revitalization” public welfare program in Hunan Province, China

**DOI:** 10.3389/fpsyg.2025.1626816

**Published:** 2025-08-29

**Authors:** Rongji Zhou, Bin Chen

**Affiliations:** Rural Industry Revitalization Research Center, College of Economics and Management, Hengyang Normal University, Hengyang, Hunan, China

**Keywords:** rural revitalization strategy, college students, willingness to participate, theory of planned behavior, motivational mechanism

## Abstract

Grounded in the Theory of Planned Behavior (TPB), this study develops a framework comprising five core constructs: rural revitalization cognition, behavioral attitude, subjective norm, perceived behavioral control, and willingness to participate. Drawing on the “Furong Scholars • Rural Revitalization” program in Hunan, China, we surveyed 634 college students and employed SEM, mediation analysis, and DEMATEL to explore the micro-level motivational mechanisms influencing their participation willingness. The key findings are as follows: (1) Individual and social factors significantly affect willingness to participate. Female students, those with urban household registration, humanities and social sciences majors, students with higher academic qualifications, and those with stronger political consciousness exhibit greater willingness to participate. (2) Rural revitalization cognition is central to the motivational system as a strong causal factor (centrality = 1.115), positively influencing behavioral attitude, subjective norm, perceived behavioral control, and participation willingness. (3) Behavioral attitude (*p* < 0.05), subjective norm (*p* < 0.001), and perceived behavioral control (p < 0.001) all significantly enhance willingness to participate, with subjective norm and perceived behavioral control showing stronger path effects. Moreover, these three variables act as key mediators between rural revitalization cognition and willingness to participate, forming crucial motivational pathways. These findings deepen the theoretical understanding of college students’ behavioral motivations in the context of rural revitalization and offer empirical insights for designing more targeted and effective policy interventions. Specifically, enhancing students’ cognition of rural revitalization, strengthening social normative support, and improving self-efficacy are identified as vital levers for increasing their willingness to engage in rural development initiatives.

## 1 Introduction

In the new era, the Rural Revitalization Strategy has emerged as a crucial initiative to address urban–rural disparities (Cai et al., 2024), modernize agriculture and rural areas, and advance China’s goal of building a modern socialist country. Human resources are pivotal for implementing this strategy, with college students representing a vital pool of high-quality talent. Their innovation, expertise, and sense of social responsibility contribute significantly to rural revitalization. The report of the 20th National Congress of the Communist Party of China explicitly calls for the “comprehensive promotion of rural revitalization” and emphasizes cultivating and guiding talent, underscoring the strategic role of human resources in driving rural development. Engaging college students in rural revitalization helps alleviate talent shortages and injects momentum into rural talent systems and socio-economic growth (Sun and Ma, 2023). However, the long-standing urban–rural dual structure has led to persistent challenges in rural areas, such as inadequate infrastructure and limited public services. Simultaneously, the willingness of high-quality college students to participate remains low (Wu et al., 2023), forming a significant bottleneck in the progress of rural revitalization (WANG and WANG, 2019). Against this backdrop, examining college students’ willingness and the underlying motivational mechanisms to engage in rural revitalization holds substantial practical significance.

Existing research has mainly explored factors influencing college students’ willingness to participate at two levels. At the individual level, studies highlight the importance of students’ awareness of rural issues (Li et al., 2021; Liu et al., 2024; Li et al., 2023), career aspirations (Xu, 2024; Peng and Hou, 2021), and practical experiences (Ju and Cai, 2024). For example, there is a significant positive correlation between students’ understanding and recognition of rural revitalization policies and their willingness to participate. Moreover, emotional attitudes and expectations of potential benefits play an influential role in decision-making (Huang, 2024). These findings demonstrate how individual cognition and emotional factors shape college students’ decisions. At the societal level, research has focused on the influence of national policy support (Wang and Zhuo, 2018; Gu, 2021), rural development conditions (Xu et al., 2024; Sun, 2024), and broader social environments (Ren and Yang, 2020; Hu, 2020; Zhan and Chen, 2020) in shaping students’ willingness to participate. Empirical studies suggest that improvements in rural infrastructure, government policies, and employment environments significantly enhance college students’ enthusiasm for rural engagement (Lei et al., 2023; Zhang et al., 2025).

While these studies provide valuable insights, they often examine internal and external factors separately and lack a unified theoretical explanation. Notably, the role of students’ cognition—especially their domain-specific understanding of rural revitalization—as an integrated psychological mechanism has been under-theorized. Wang and He (2023) emphasized the importance of rural policy understanding in enhancing participation willingness. However, their study does not fully explain how such understanding transforms into intention via psychological mechanisms. This gap provides an opportunity to advance theory by embedding rural revitalization cognition into an established behavioral framework.

The existing literature has contributed to a relatively comprehensive understanding of rural engagement. However, we identify several areas where further investigation is warranted. First, a more systematic integration of individual cognition and environmental factors is needed, as these elements interact dynamically in real-world contexts. Second, quantitative methods such as mediation analysis or structural equation modeling (SEM) remain underutilized. Third, most prior models assume behavior is guided mainly by rational evaluation, with less attention to experiential or domain-specific knowledge.

To address these gaps, this study extends the Theory of Planned Behavior (TPB) by incorporating “rural revitalization cognition” as a context-specific antecedent. This construct reflects students’ understanding, interpretation, and experiential engagement with rural development strategies. Its inclusion provides explanatory power beyond standard TPB components such as attitude or perceived control, offering a more holistic model of motivation. Moreover, this framework responds to common critiques of TPB—such as its over-reliance on rational reasoning and the well-known intention-behavior gap (Sniehotta et al., 2014; Sheeran, 2002)—by incorporating cognition as a mediating foundation that connects exposure with intention. Methodologically, this study employs a mixed-methods approach combining SEM, mediation analysis, and the Decision-Making Trial and Evaluation Laboratory (DEMATEL) technique to map out the influence pathways among cognition, attitudes, norms, perceived control, and willingness.

The study’s main contribution lies in extending TPB both theoretically and empirically by integrating rural revitalization cognition and quantitatively validating the model using robust statistical tools. This provides novel insights for mobilizing college students in rural development and offers practical implications for policy and educational interventions.

## 2 Materials and research methods

### 2.1 Theoretical analysis and research hypotheses

**Figure 1:**
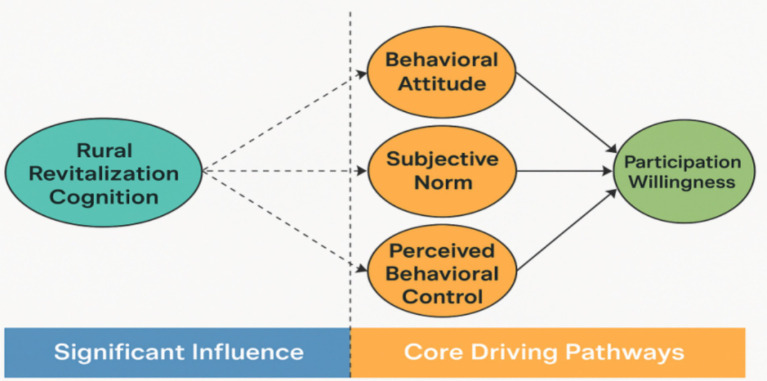
Theoretical analysis framework.

Integrating the Theory of Planned Behavior (TPB) with rural revitalization cognition, this study proposes a comprehensive theoretical framework to examine college students’ willingness to participate in rural revitalization(See Figure 1). Developed by Ajzen, TPB explains how individuals form behavioral intentions and how these intentions translate into actual behavior (Sungur-Gül and Ateş, 2021; Hagger et al., 2022). As a widely applied model across disciplines, TPB has demonstrated substantial explanatory power in fields such as education and economics. Accordingly, TPB serves as the primary framework for understanding college students’ participation intentions in this study. According to TPB, behavioral intention is shaped by three core factors: behavioral attitude, subjective norm, and perceived behavioral control (Su et al., 2021). Behavioral attitude reflects an individual’s evaluation of a behavior and its potential outcomes, including perceived benefits or drawbacks (Ajzen, 2020). Subjective norm captures the perceived expectations and pressures from significant others or social groups, such as family, friends, teachers, or government authorities (Sussman and Gifford, 2019). Perceived behavioral control concerns an individual’s assessment of their capability to perform a behavior, including perceived feasibility and difficulty (La Barbera and Ajzen, 2021).

To extend the TPB framework contextually, this study introduces “rural revitalization cognition” as a domain-specific cognitive construct that captures students’ awareness, knowledge, and experience regarding rural revitalization strategies, programs, and initiatives. Unlike general attitudes or perceived control, this construct focuses on the interpretive and experiential understanding derived from policy exposure, practical engagement, and social discourse. This theoretical extension is grounded in social cognitive theory and experiential learning literature, which emphasize that behavior is not only driven by rational appraisal but also by prior knowledge structures and contextual meaning-making (Sereenonchai et al., 2020). By integrating this cognition into TPB, the model better explains how students’ informed understanding can shape their perceptions of desirability, normative expectations, and feasibility regarding rural participation. Additionally, this extension responds to well-documented critiques of TPB. Scholars such as Sniehotta et al. (2014) and Sheeran (2002) have argued that TPB overly emphasizes deliberate reasoning, overlooking affective and unconscious drivers and the well-known intention-behavior gap. By including rural revitalization cognition as a foundation, this model bridges knowledge with motivation and indirectly addresses the gap between knowing and doing.

In this study, college students’ cognition of rural revitalization—including their awareness of related strategies, volunteer projects, and public welfare initiatives—plays a crucial role in shaping behavioral attitude, subjective norm, and perceived behavioral control. Behavioral attitude is the first component of the TPB model and directly influences behavioral intention. TPB suggests that individuals are more inclined to engage in a behavior when they hold a positive attitude toward its outcomes. In the rural revitalization context, students who perceive participation as beneficial to personal growth, family wellbeing, or societal development are more likely to express strong intentions to participate. Subjective norm refers to perceived social pressure to perform a behavior. TPB posits that individuals are likelier to engage in behaviors endorsed or expected by significant others. In rural revitalization, college students’ perceptions of government initiatives, family encouragement, or peer support can substantially enhance their willingness to participate. Perceived behavioral control reflects an individual’s sense of ease or difficulty in undertaking a behavior. When students feel equipped with sufficient resources and support to engage in rural revitalization, their intention to participate strengthens. Conversely, perceptions of barriers or resource constraints may diminish their willingness to act.

As pointed out by Wang and He (2023), students’ understanding of rural revitalization policies plays a positive role in shaping participation intentions. However, few studies have integrated this domain-specific cognition systematically into TPB or explored its underlying psychological pathways. This study builds on their findings by conceptualizing rural cognition as a core antecedent that activates TPB components. College students’ cognition of rural revitalization stems from exposure to information, practical engagement, coursework, and policy advocacy. This cognitive level reflects both their understanding and their experiential depth, making it a distinctive experiential variable. Drawing on the theoretical perspective that “richer experiences significantly promote the adoption of effective practices” (Sereenonchai et al., 2020), this study proposes that rural revitalization cognition influences behavioral intention through a sequential psychological mechanism. Specifically, enhanced cognition can first improve behavioral attitude (ATT) by deepening students’ understanding of participation benefits. Second, it can reinforce subjective norm (SUB), increasing sensitivity to policy directions and societal expectations, thus amplifying social influence. Third, it can strengthen perceived behavioral control (PER) by clarifying available resources and implementation pathways, reducing perceived barriers, and enhancing self-efficacy. Collectively, ATT, SUB, and PER serve as core mediators through which cognition affects participation intention, forming a progressive chain of “expected evaluation – norm internalization – perceived efficacy.” As students’ cognitive levels rise, their optimism about participation outcomes, responsiveness to social expectations, and confidence in executing actions synergize, fostering a positively evolving psychological system that significantly boosts sustainable behavioral intentions toward rural revitalization.

Based on these theoretical insights, the following hypotheses are proposed:

*H1*: College students’ cognition of rural revitalization positively influences their behavioral attitudes, subjective norms, and perceived behavioral control.

*H2*: College students’ behavioral attitudes, subjective norms, and perceived behavioral control positively influence their willingness to participate in rural revitalization.

*H3*: College students’ cognition of rural revitalization significantly enhances their willingness to participate, with behavioral attitude, subjective norm, and perceived behavioral control acting as mediators in this process.

### 2.2 Questionnaire design and data sources

Building on the theoretical framework and research hypotheses described above, this study follows Ajzen’s guidelines for questionnaire design and draws on existing literature, using the “Furong Scholars • Rural Revitalization” public welfare program in Hunan Province as a representative case. A survey was conducted among students from several universities in Hunan Province. A total of 650 questionnaires were distributed, with 634 valid responses collected, resulting in an effective response rate of 97.54%. The questionnaire comprises two main sections. The first section collects information on respondents’ individual and social characteristics, including gender, household registration, university type, major, and political affiliation. These variables will serve as instrumental variables in subsequent analyses. The second section contains scale-based items designed to measure the study’s key variables: rural revitalization cognition, behavioral attitude, subjective norm, perceived behavioral control, and participation intention. This section employs a five-point Likert scale ranging from 1 (“Strongly Disagree”) to 5 (“Strongly Agree”) to capture responses. In total, the scale section includes 18 items (detailed in Table 1), which assess students’ levels of rural revitalization cognition as well as their behavioral attitudes, subjective norms, perceived behavioral control, and willingness to participate.

**Table 1:** Scale design and questionnaire items.

Dimension	Code	Item	Options
participation Willingness	INT_1_	I am willing to participate in the “Sanxiaxiang” or “Furong Scholars” public welfare programs.	1 = Strongly Disagree, 2 = Disagree, 3 = Neutral, 4 = Agree, 5 = Strongly Agree
	INT_2_	I will try or continue to participate in the “Sanxiaxiang” or “Furong Scholars” public welfare programs in the future.	
	INT_3_	I am willing to share my experience in the “Sanxiaxiang” or “Furong Scholars” public welfare programs with others.	
Rural revitalization cognition	COG_1_	My understanding of China’s rural revitalization strategy.	
	COG_2_	My understanding of volunteer programs such as the Western Volunteer Program and rural assistance initiatives.	
	COG_3_	My awareness of public welfare programs such as “Sanxiaxiang” and “Furong Scholars” in Hunan Province.	
behavioral Attitude	ATT_1_	I am interested in the “Sanxiaxiang” or “Furong Scholars” public welfare programs.	
	ATT_2_	Participating in these programs helps me realize my self-worth.	
	ATT_3_	Participation in these programs enables me to gain practical experience and develop hands-on skills.	
	ATT_4_	Participation in these programs allows me to gain recognition from others (e.g., villagers, volunteer organizations).	
	ATT_5_	Participating in these programs contributes to the development and revitalization of rural areas in China.	
Subjective norms	SUB_1_	Policy support from the government and universities for participation in “Furong Scholars” programs motivates me to engage in rural revitalization volunteer activities.	
	SUB_2_	My parents/family members’ proactive involvement in helping others motivates me to participate in these programs.	
	SUB_3_	Encouragement from my university to engage in volunteer service motivates me to participate.	
	SUB_4_	Seeing my friends actively participate in large-scale volunteer activities motivates me to join.	
Perceived behavioral control	PER_1_	Participating in rural public welfare activities is easy for me.	
	PER_2_	I have the necessary knowledge and skills to participate in these programs.	
	PER_3_	I have sufficient physical and mental energy to engage in these programs.	

### 2.3 Sample characteristics analysis

Descriptive statistics were conducted on the survey data to analyze respondents’ characteristics (see Table 2). Overall, the sample distribution closely reflects the typical demographics of college students in Hunan Province. Regarding willingness to participate in rural revitalization, 215 respondents reported “agree” and 221 “strongly agree,” together accounting for 68.8% of the sample. This indicates that most students expressed strong intentions to engage actively in rural revitalization efforts.

**Table 2:** Basic information of the sample.

Variable	Category	Frequency	Percentage (%)	Variable	Category	Frequency	Percentage (%)
Gender	Male	202	31.9	Major type	Humanities and social sciences	464	73.2
	Female	432	68.1		Natural sciences	159	25.1
Household registration	Rural	455	71.8	Political status	Communist party member	113	17.8
	Urban	179	28.2		Communist youth league member	379	59.8
University type	Double first-class	86	13.6		Non-affiliated	142	22.4
	General universities	438	69.1	Participation willingness	Agree	215	33.9
	Vocational colleges	110	17.4		Strongly agree	221	34.9

Previous studies have shown that college students’ willingness to participate is shaped by a combination of individual and social factors (Wang and He, 2023; Zhong and Liu, 2019; Wang and Zhao, 2024). To examine these effects, this study included five control variables: gender, household registration, university type, major, and political status. Cross-tabulations and chi-square tests were performed to assess significant differences between groups, with results presented in Table 3. Significant differences were observed based on university type, major, and political status. The key findings are summarized as follows:

**Table 3:** Test results of group differences.

Category	Chi-square value	Degrees of freedom	*p*-value
Gender	6.622	4	0.157
Household registration	5.379	4	0.251
University type	80.663	8	0.000^***^
Major type	22.228	12	0.035^*^
Political status	29.474	8	0.000^***^

***P < 0.001, **P < 0.05, *P < 0.01, indicating statistical significance levels for Chi-square test between groups.

Gender: Among male students, 62.87% indicated “agree” or “strongly agree,” compared to 71.53% of female students, suggesting higher willingness among females. However, the chi-square test did not identify statistically significant gender differences.

Household Registration: Willingness to participate was reported by 67.03% of rural students and 73.18% of urban students, implying higher engagement among urban students, though differences were not statistically significant.

University Type: Among different types of universities, 48.84% of students from Double First-Class universities, 78.77% from regular universities, and 44.55% from vocational colleges expressed “agree” or “strongly agree.” These results indicate a positive relationship between higher educational levels (undergraduate > vocational) and willingness to participate, with significant differences confirmed by the chi-square test. This aligns with prior research suggesting that “a higher level of education is associated with a greater tendency to adopt positive thinking” (Sereenonchai and Arunrat, 2019). In other words, students with higher educational backgrounds are more capable of comprehensive and positive evaluations regarding participation in rural revitalization, thereby enhancing their willingness to engage.

Major: Among students from different academic disciplines, 72.20% of humanities and social sciences majors and 57.23% of natural sciences majors reported “agree” or “strongly agree,” indicating higher willingness among humanities and social sciences students. Significant differences were found between these groups.

Political Status: Regarding political affiliation, 61.06% of Communist Party members, 73.88% of Communist Youth League members, and 61.27% of non-affiliated students expressed “agree” or “strongly agree.” These results suggest that Communist Youth League members exhibit significantly higher willingness to participate, while Party members and non-affiliated students show similar levels of willingness. Significant differences were detected by the chi-square test.

### 2.4 Research methods

#### 2.4.1 Structural equation modeling (SEM)

This study investigates the pathways through which multiple factors influence college students’ willingness to participate in rural revitalization. SEM was chosen over alternative methods such as multiple regression or path analysis due to its ability to model latent constructs, account for measurement error, and simultaneously test complex mediation and interaction effects within a unified framework (Hedges and Olkin, 2016). Unlike traditional regression techniques, SEM provides the flexibility to model both direct and indirect relationships between latent variables and their observed indicators, which is essential for exploring multi-dimensional constructs like cognition, attitude, and intention in this study.

The SEM framework comprises two key parts: the measurement model and the structural model. The measurement model describes the relationships between latent variables and observed variables, expressed as:


$$\underset{q\times 1}{X}=\underset{q\times n}{\mathrm{\Lambda x}}\underset{n\times 1}{\xi }+\underset{q\times 1}{\delta }$$

$$\underset{p\times 1}{Y}=\underset{p\times m}{\mathrm{\Lambda y}}\underset{m\times 1}{\eta }+\underset{p\times 1}{\varepsilon }$$

In this equation, ξ represents the exogenous latent variable, and η represents the endogenous latent variable. X and Y are the observed indicators for ξ and η, respectively. δ and ε are the corresponding measurement errors. Λx is the factor loading q×n -order matrix that reflects the relationship between the observed indicators X and the exogenous latent variable ξ; Λy is the factor loading p×m -order matrix that represents the relationship between the observed indicators Y and the endogenous latent variable η. m and n represent the number of endogenous and exogenous latent variables, while p and q indicate the number of corresponding observed indicators.

The structural model is used to describe the interrelationships between latent variables. Its expression is as follows:


$$\underset{m\times 1}{\eta }=\underset{m\times m}{B}\underset{m\times 1}{\eta }+\underset{m\times n}{\Gamma }\underset{n\times 1}{\xi }+\underset{m\times 1}{\varsigma }$$

In this equation, ς represents the random error term, and η reflects the unexplained part of the endogenous latent variable. B is the path coefficient matrix of endogenous latent variables, which is used to depict the interactive relationships between endogenous latent variables. Γ is the path coefficient matrix of exogenous latent variables, representing the effect of exogenous latent variables on endogenous latent variables.

This structural model construction is informed by Hedges and Olkin (2016), who provided a framework that allows for the simultaneous consideration of both direct and indirect paths, making it suitable for testing complex mediation and interaction effects.

#### 2.4.2 Mediation model

To test whether COG positively influences INT and whether ATT, SUB, and PER act as mediators, two complementary methods were employed: stepwise regression and the Bootstrap method, enhancing result robustness.

Stepwise regression is used to sequentially introduce variables into the model based on their explanatory power. In this study, the model starts with COG as the main predictor and gradually includes ATT, SUB, and PER to examine their mediating roles between COG and INT.

Model specifications:


$${\mathrm{INT}}_{i}=\beta_{0}+\beta_{1}{\mathrm{COG}}_{i}+\sum_{j=1}^{5}\gamma_{j}{\text{Tool}}_{\mathrm{ij}}+\varepsilon_{i}$$

$${\mathrm{INT}}_{i}=\beta_{0}+\beta_{1}{\mathrm{COG}}_{i}+\beta_{2}{\mathrm{ATT}}_{i}+\sum_{j=1}^{5}\gamma_{j}{\text{Tool}}_{\mathrm{ij}}+\varepsilon_{i}$$

$${\mathrm{INT}}_{i}=\beta_{0}+\beta_{1}{\mathrm{COG}}_{i}+\beta_{2}{\mathrm{ATT}}_{i}+\beta_{3}{\mathrm{SUB}}_{i}+\beta_{4}{\mathrm{PER}}_{i}+\sum_{j=1}^{5}\gamma_{j}{\text{Tool}}_{\mathrm{ij}}+\varepsilon_{i}$$

$${\mathrm{INT}}_{i}=\beta_{0}+\beta_{1}{\mathrm{COG}}_{i}+\beta_{2}{\mathrm{ATT}}_{i}+\beta_{3}{\mathrm{SUB}}_{i}+\sum_{j=1}^{5}\gamma_{j}{\text{Tool}}_{\mathrm{ij}}+\varepsilon_{i}$$

Among them, Tool represents the instrumental variables (gender, household registration, school type, major category, and political affiliation), which are used to control for individual background differences.

The Bootstrap method is a non-parametric resampling approach widely used for estimating indirect (mediating) effects and their confidence intervals. In this study, Bootstrap is used to test whether ATT, SUB, and PER mediate the relationship between COG and INT. Procedure:

Step 1: COG → Mediator (M)


$$M_{i}=\alpha_{0}+\alpha_{1}{\mathrm{COG}}_{i}+\sum_{j=1}^{5}\delta_{j}{\text{Tool}}_{\mathrm{ij}}+u_{i}$$

Step 2: Mediator and COG → INT


$${\mathrm{INT}}_{i}=\theta_{0}+\theta_{1}{\hat{M}}_{i}+\theta_{2}{\mathrm{COG}}_{i}+\sum_{j=1}^{5}\lambda_{j}{\text{Tool}}_{\mathrm{ij}}+v_{i}$$

Bootstrap resampling (1,000 iterations): The above steps are repeated 1,000 times to generate a sampling distribution of the indirect effect $\theta_{1}$ , from which the mean and 95% confidence interval are calculated.

The use of the Bootstrap method and its model construction follows the methodology outlined by Preacher and Hayes (2008), which emphasizes the importance of resampling and asymptotic methods for assessing indirect effects.

#### 2.4.3 Decision trial and evaluation laboratory method (DEMATEL)

DEMATEL analyzes complex systems using matrix operations and graph theory (Li and Sun, 2019). This method was selected because of its strengths in revealing causal relationships and prioritizing influencing factors, especially in systems where feedback loops and interdependencies are present. Compared to methods like ANP (Analytic Network Process) or ISM (Interpretive Structural Modeling), DEMATEL offers a more intuitive visualization of factor influence strength and direction, which is particularly suitable for identifying key leverage points in policy or decision contexts such as rural revitalization engagement.

The calculation process is as follows:

First, construct the direct influence matrix Z as follows:


$$Z={\left(z_{\mathrm{ij}}\right)}_{n\times n}$$

Next, standardize the direct influence matrix to obtain the normalized influence matrix G as follows:


$$G={\left(I-\mathrm{\alpha Z}\right)}^{-1}$$

Then, calculate the total influence matrix T by using the identity matrix I:


T=G·Z

Finally, based on the total influence matrix T, compute the influence degree $R_{i}$ , affected degree $E_{i}$ , centrality $C_{i}$ , and causality $Y_{i}$ for each factor as follows:


$$R_{i}=\sum_{j=1}^{n}t_{\mathrm{ij}},\;\;E_{i}=\sum_{j=1}^{n}t_{\mathrm{ji}},\;\;C_{i}=R_{i}+E_{i},\;\;Y_{i}=R_{i}-E_{i}$$

These calculations allow for the identification of key factors that influence the overall system and help prioritize interventions (Tzeng et al., 2007). The model construction for DEMATEL and its application in analyzing complex systems is based on the hybrid MCDM model proposed by Tzeng et al. (2007), which uses factor analysis and DEMATEL to analyze causal relationships in multi-criteria decision-making.

#### 2.4.4 Semi-structured interviews

Semi-structured interviews were developed based on the study’s questionnaire and relevant literature on college students’ engagement in rural revitalization. The interviews aimed to gain deeper insights into students’ attitudes toward rural revitalization and explore factors influencing their willingness to participate. Each interview lasted approximately 20 min and followed a guided conversational format, with detailed notes documented throughout.

## 3 Results and analysis

### 3.1 Reliability and validity testing

This study evaluated the scale’s quality in terms of reliability and validity. The scale comprises 18 items, and the analysis results are presented in Table 4. The overall Cronbach’s *α* coefficient is 0.962, indicating excellent internal consistency. Specifically, the reliability coefficients for the five subscales—rural revitalization cognition, behavioral attitude, subjective norm, perceived behavioral control, and participation willingness—are 0.870, 0.914, 0.860, 0.867, and 0.887, respectively. All exceed the commonly accepted threshold of 0.6, demonstrating strong reliability for each dimension. Regarding validity, the overall KMO (Kaiser-Meyer-Olkin) value of the scale is 0.971, indicating sampling adequacy for factor analysis. The KMO values for the five subscales range from 0.733 to 0.894, all above the recommended minimum of 0.6. Further analysis shows that the communalities for all items exceed 0.6, confirming the appropriateness of the scale design. Factor analysis reveals that the 18 items load onto five distinct factors, with a cumulative variance explanation of 78.158%, substantially surpassing the 50% benchmark. Moreover, all items exhibit factor loadings above 0.7, indicating a strong correspondence between items and their respective latent variables and aligning well with the theoretical framework.

**Table 4:** Reliability and validity test results of the scale.

Scale	Items	Reliability		Validity		
		α coefficient		KMO		Factor loadings
Intention (INT)	INT_1_, INT_2_, INT_3_	0.962	0.887	0.971^***^	0.745^***^	0.908, 0.908, 0.891
Rural revitalization cognition (COG)	COG_1_, COG_2_, COG_3_		0.870		0.733^***^	0.869, 0.902, 0.902
Behavioral attitude (ATT)	ATT_1_, ATT_2_, ATT_3_, ATT_4_, ATT_5_		0.914		0.894^***^	0.818, 0.883, 0.884, 0.870, 0.861
Subjective norm (SUB)	SUB_1_, SUB_2_, SUB_3_, SUB_4_		0.860		0.811^***^	0.883, 0.742, 0.868, 0.857
Perceived BEHAVIORAL Control (PER)	PER_1_, PER_2_, PER_3_		0.867		0.735^***^	0.874, 0.893, 0.901

***P < 0.001, **P < 0.05, *P < 0.01, indicating statistical significance levels for Chi-square test between groups.

In addition to evaluating the structural model’s reliability and sampling adequacy using Cronbach’s α and the Kaiser-Meyer-Olkin (KMO) measure, this study also assesses the model’s construct validity through the latent variable correlation matrix and the square roots of the Average Variance Extracted (AVE), as presented in Table 5.

**Table 5:** Discriminant validity: Pearson correlation and the square root of AVE.

Variable	COG	ATT	SUB	PER	INT
COG	**0.876432**	0.468798	0.450567	0.580338	0.549005
ATT	0.468798	**0.832804**	0.617539	0.562899	0.638706
SUB	0.450567	0.617539	**0.80249**	0.545551	0.597055
PER	0.580338	0.562899	0.545551	**0.868738**	0.626872
INT	0.549005	0.638706	0.597055	0.626872	**0.885441**

The bold values on the diagonal represent the square root of the AVE.

First, convergent validity examines how well a latent variable explains the variance of its indicators. Following the Fornell-Larcker criterion, an AVE value above 0.5 indicates adequate convergence. In this study, all latent variables meet this standard: COG has an AVE of 0.768, ATT 0.694, INT 0.784, PER 0.755, and SUB 0.644. These results demonstrate strong convergent validity for each construct, confirming that the latent variables effectively capture the variance of their respective indicators.

Second, discriminant validity evaluates whether latent variables are distinct from one another. The Fornell-Larcker criterion requires that the square root of each latent variable’s AVE exceeds its correlations with other latent variables. The results show that the square roots of the AVE for COG (0.876), ATT (0.832), INT (0.885), and other constructs are all greater than their inter-construct correlations—for example, the correlation between COG and ATT is 0.469. This indicates that each latent variable is conceptually distinct and adequately separated from others, satisfying discriminant validity requirements.

Finally, the correlation matrix reveals moderate correlations among latent variables, with no excessively high values that would indicate multicollinearity. For instance, the correlations between COG and ATT (0.469), COG and SUB (0.451), and INT and PER (0.627) suggest reasonable associations without problematic overlap. These moderate correlations further support the model’s good discriminant validity.

Therefore, the structural model in this study demonstrates robust construct validity, providing a solid foundation for subsequent analyses and path testing.

### 3.2 SEM model results analysis

Based on the theoretical framework and results of the reliability and validity tests, this study constructed a structural equation model (SEM) to examine the relationships among latent and observed variables. Model estimation and fit analyses were conducted using AMOS software. All fit indices met the predetermined thresholds (see Table 6), indicating a well-fitting and robust model.

**Table 6:** SEM model fit results.

Fit Index		Criteria	Value
Absolute indices	χ^2^/df	<5	3.292
	GFI	>0.8	0.930
	RMSEA	<0.1	0.060
	RMR	<0.08	0.040
	SRMR	<0.1	0.037
	AGFI	>0.8	0.904
Relative indices	CFI	>0.8	0.969
	NFI	>0.8	0.956
	NNFI	>0.8	0.962
	IFI	>0.8	0.969
	TLI	>0.8	0.962
Parsimony indices	PGFI	>0.5	0.680
	PNFI	>0.5	0.781

**Figure 2:**
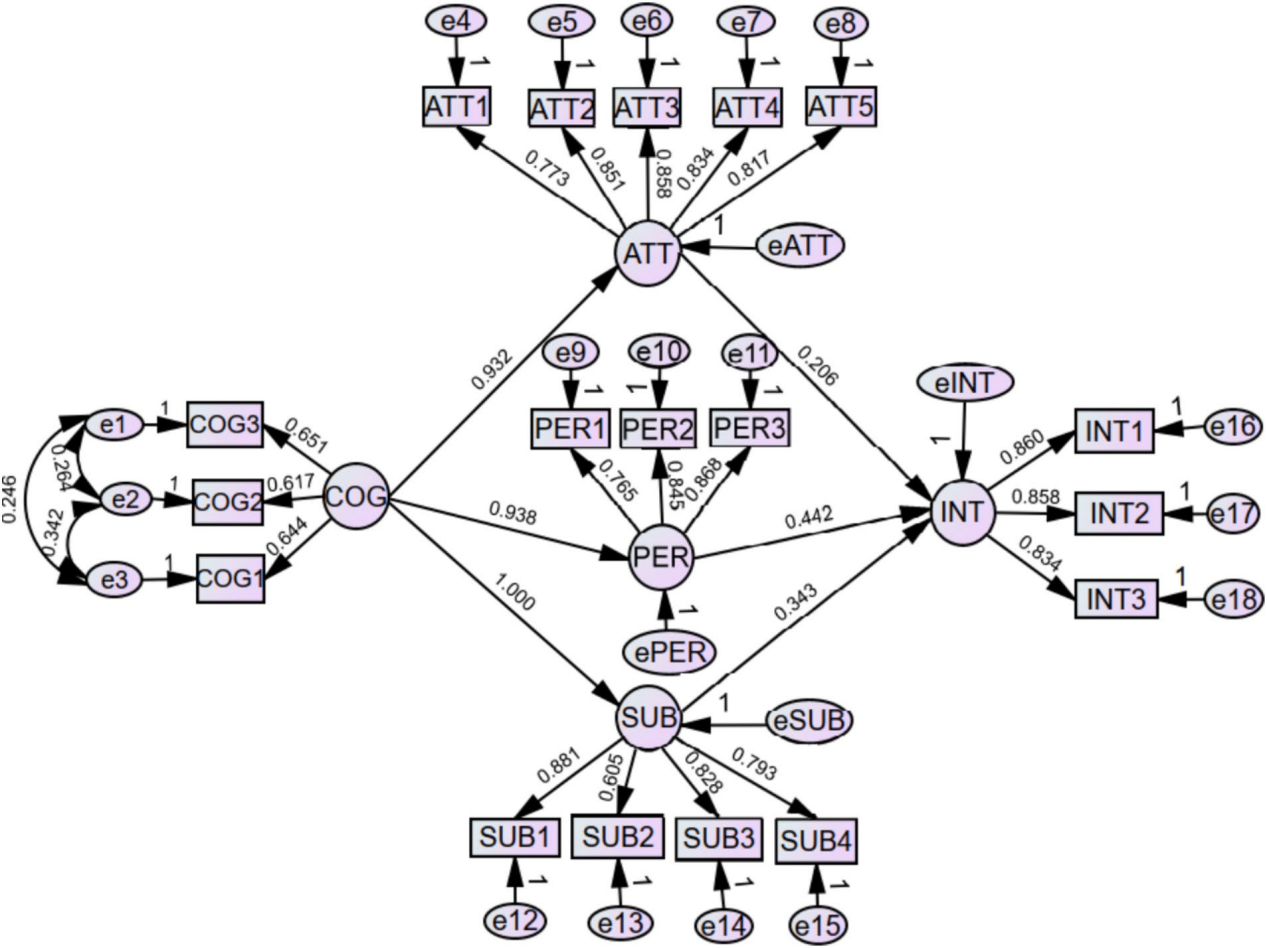
Structural equation model.

The structural model tested the TPB hypotheses, with results presented in Table 7 and Figure 2. Findings reveal that “behavioral attitude,” “subjective norm,” and “perceived behavioral control” constitute the primary pathways influencing college students’ willingness to participate in rural revitalization, jointly explaining approximately 90% of the variance in participation willingness (*R*^2^ = 0.899). Additionally, “rural revitalization cognition” significantly impacts these three mediators. The detailed results are as follows:

**Table 7:** Model path estimation results.

Path	Unstandardized path coefficient	C. R	Standardized path coefficient
COG → ATT	1.12	16.648	0.932^***^
COG → SUB	1.358	18.081	1.000^***^
COG → PER	1.154	15.582	0.938^***^
ATT → INT	0.225	1.994	0.206^*^
SUB → INT	0.333	3.836	0.343^***^
PER → INT	0.474	7.197	0.442^***^
COG → COG_3_	1.013	19.696	0.644^***^
COG → COG_2_	0.931	19.229	0.617^***^
COG → COG_1_	1	-	0.651^***^
ATT → ATT_5_	0.996	22.482	0.817^***^
ATT → ATT_4_	1.022	23.054	0.834^***^
ATT → ATT_3_	1.032	23.922	0.858^***^
ATT → ATT_2_	1.023	23.664	0.851^***^
ATT → ATT_1_	1	-	0.773^***^
SUB → SUB_4_	0.896	25.553	0.793^***^
SUB → SUB_3_	0.947	27.555	0.828^***^
SUB → SUB_2_	0.546	17.003	0.605^***^
SUB → SUB_1_	1	-	0.881^***^
PER → PER_3_	1.078	23.082	0.868^***^
PER → PER_2_	1.011	22.405	0.845^***^
PER → PER_1_	1	-	0.765^***^
INT → INT_3_	0.953	26.913	0.834^***^
INT → INT_2_	1	28.288	0.858^***^
INT → INT_1_	1	-	0.860^***^

***P < 0.001, **P < 0.05, *P < 0.01, indicating statistical significance levels for Chi-square test between groups.

First, all observed variables—COG1 to COG3; ATT1 to ATT5; SUB1 to SUB4; PER1 to PER3; and INT1 to INT3—exhibited significant factor loadings on their corresponding latent constructs: rural revitalization cognition (COG), behavioral attitude (ATT), subjective norm (SUB), perceived behavioral control (PER), and participation intention (INT). These results confirm strong construct validity, demonstrating that the indicators effectively represent their theoretical dimensions.

Second, the standardized path coefficients from COG to ATT, SUB, and PER were 0.932, 1.000, and 0.938, respectively (*p* < 0.001), indicating significant and substantial positive effects and supporting Hypothesis H1. Rural revitalization cognition encompasses students’ understanding of the strategic importance, policy implications, and societal impact of rural revitalization efforts, including volunteer programs and public welfare initiatives. This cognitive dimension critically shapes the formation of the core TPB constructs and serves as a foundational driver of behavioral intention.

To supplement this quantitative finding, interview participants frequently emphasized that gaining a deeper understanding of rural revitalization—especially through school-organized talks, rural service project briefings, and media exposure—was crucial to shaping their perception. One student noted, “After learning how rural revitalization aligns with national development, I felt more responsible to be involved.” These qualitative insights affirm that cognition is not merely a background factor but a key activator of psychological mechanisms in the TPB framework.

Third, the standardized path coefficient from ATT to INT was 0.206 (*p* < 0.05), confirming a significant positive relationship and supporting Hypothesis H2. Behavioral attitude reflects students’ evaluations of participating in rural revitalization, encompassing both affective and cognitive appraisals. These attitudes assess whether participation aligns with personal interests, fosters self-development, enhances skills, promotes social recognition, and contributes to rural and national development, serving as essential predictors of behavioral intention under TPB.

Students also expressed in interviews that their positive attitudes stemmed from real-life stories shared by peers and alumni who had previously participated in such programs. These narratives often conveyed personal growth, a sense of fulfillment, and stronger community bonds, further reinforcing favorable attitudes.

Fourth, the standardized path coefficient from SUB to INT was 0.343 (*p* < 0.001), indicating a significant positive effect and validating Hypothesis H2. Subjective norm captures perceived social expectations from significant referents such as family, peers, educators, or government entities regarding rural revitalization participation. As Marx observed, “The human essence is the ensemble of social relations” (Wang and Hou, 2024). During university years, when students are highly receptive to social influences, guidance and encouragement from key social actors can significantly shape behavioral intentions.

This is consistent with interview findings where students reported that the opinions of teachers and parents often served as a tipping point in deciding whether to join such projects. For example, a participant shared, “My tutor encouraged me, saying it’s a good opportunity for both career development and giving back to society.”

Fifth, the standardized path coefficient from PER to INT was 0.442 (*p* < 0.001), demonstrating a strong and significant positive effect, supporting Hypothesis H2. Perceived behavioral control reflects students’ perceptions of their capacity to engage in rural revitalization, encompassing feasibility and anticipated challenges. From a theoretical perspective, practice represents the transformation of knowledge into action, engaging individuals in shaping the social world. Students’ judgments about their competencies—including their knowledge, skills, resources, and ability to manage potential social consequences—directly influence their willingness to participate (Hung and Liu, 2024).

Interviews revealed that logistical and institutional support from universities (e.g., transportation, subsidies, training) significantly boosted students’ confidence. One student remarked, “I would not have dared to go without clear support, but the school handled everything—so I said yes.”

### 3.3 Analysis of mediation effect test results

To test Hypothesis H3—that college students’ cognition of rural revitalization (COG) significantly influences their participation intention (INT), with attitude (ATT), subjective norms (SUB), and perceived behavioral control (PER) serving as mediators—this study employed both stepwise regression and the bootstrap method for a comprehensive analysis (see Tables 8, 9 for details).

**Table 8:** Stepwise regression results.

Variable	*R*-squared	Adj. *R*-squared	COG coefficient	COG *p*-value
COG only	0.478	0.473	0.68	0
+ ATT	0.728	0.725	0.296	0
+ SUB	0.745	0.742	0.261	0
+ATT, SUB, PER	0.765	0.761	0.157	0

**Table 9:** Bootstrap estimates results.

Mediation variable	Estimate	CI lower (2.5%)	CI upper (97.5%)
ATT	0.613	0.503	0.716
SUB	0.624	0.496	0.735
PER	0.806	0.643	0.963

First, stepwise regression results show that in the baseline model containing only COG, the explanatory power for INT is *R*^2^ = 0.478, with a significant COG coefficient of 0.680 (*p* = 0.000). As mediators are sequentially added, model fit improves: adding ATT raises *R*^2^ to 0.728, and the COG coefficient drops to 0.296; with SUB included, *R*^2^ increases to 0.745, and the COG coefficient decreases further to 0.261; finally, incorporating PER raises *R*^2^ to 0.765, with the COG coefficient reduced to 0.157, though still significant (*p* = 0.000). This trend suggests that while mediators reduce the direct effect of COG on INT, the effect remains significant, indicating partial mediation. The substantial decline in the COG coefficient from 0.680 to 0.157—while remaining statistically significant—suggests that the influence of cognition on intention is largely transmitted through the mediators rather than exerted directly. This highlights the central role of psychological mechanisms in translating cognitive understanding into motivational outcomes. In practice, this means that simply increasing students’ knowledge or awareness of rural revitalization may not be sufficient to generate high levels of participation intention unless it is accompanied by interventions that shape their attitudes, perceived norms, and control beliefs. Moreover, the persistence of a significant direct path from COG to INT, despite the inclusion of all mediators, underscores the complexity of this relationship. While partial mediation confirms that psychological factors serve as important transmission channels, the remaining direct effect suggests that cognitive factors may also influence intention through additional, unmeasured mechanisms—such as value alignment, emotional resonance, or contextual familiarity. This limitation calls for future research to further explore other potential mediators or moderators that contribute to this pathway.

Second, the bootstrap analysis provides further validation. The estimated indirect effects through ATT, SUB, and PER are 0.613 (95% CI: [0.503, 0.716]), 0.624 (95% CI: [0.496, 0.735]), and 0.806 (95% CI: [0.643, 0.963]), respectively. All confidence intervals exclude zero, confirming that these mediation effects are statistically significant.

Overall, results from both methods indicate that ATT, SUB, and PER significantly mediate the relationship between COG and INT. However, COG retains a significant direct effect on INT even after accounting for these mediators, indicating a partial mediation model. Thus, college students’ cognition of rural revitalization influences their participation intentions both directly and indirectly through psychological pathways such as attitudes, norms, and perceived control, highlighting the multifaceted nature of the cognition–intention relationship.

### 3.4 DEMATEL causal relationship results

**Figure 3:**
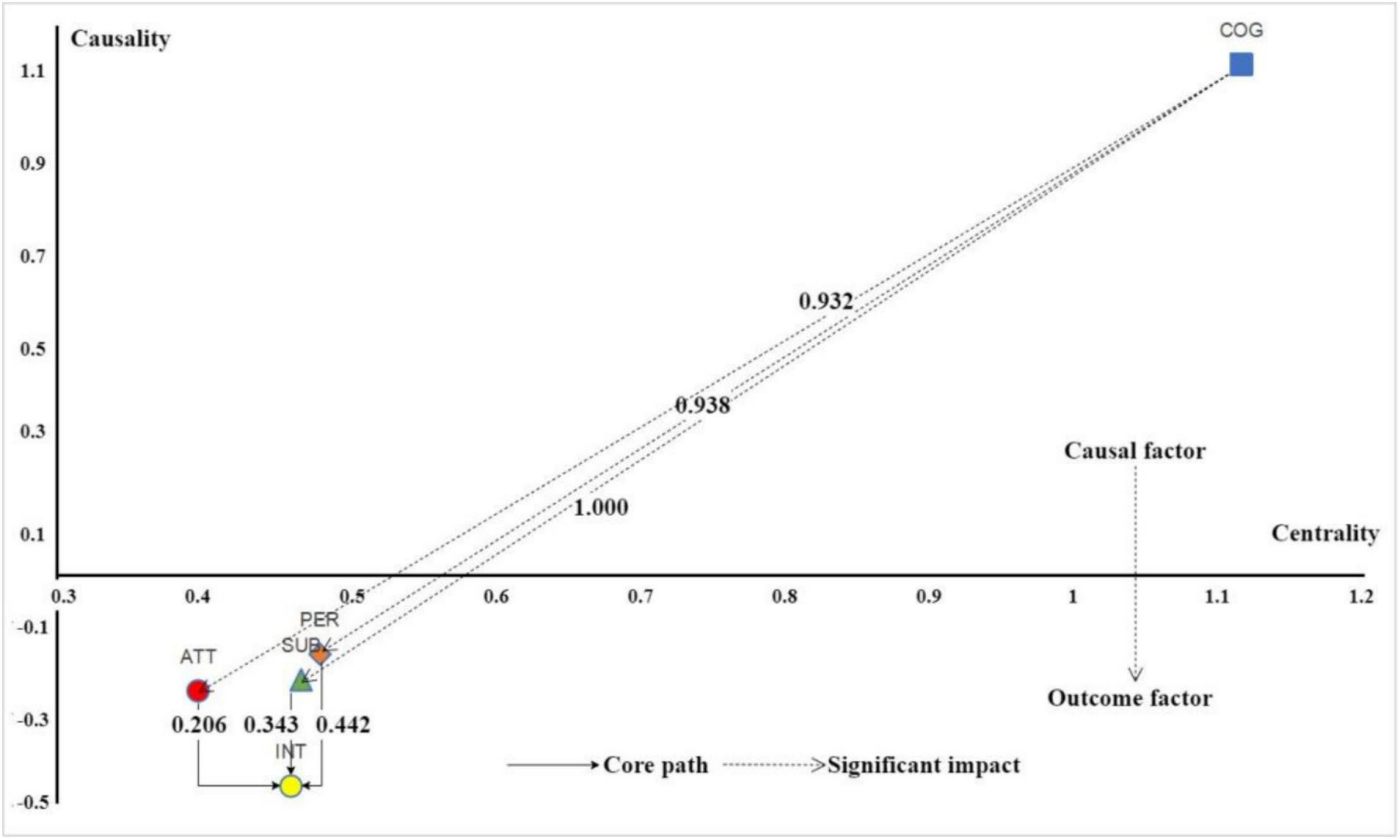
DEMATEL causal relationship diagram.

To further explore the interrelationships among variables, this study applied DEMATEL analysis by incorporating the standardized path coefficients from SEM as parameters in the standardized direct influence matrix, following the approach proposed by Guo et al. (2024). This method enabled calculation of influence paths and examination of systemic relationships (see Table 10 and Figure 3). Specifically, the DEMATEL method calculates a total influence matrix by summing direct and indirect influence values derived from the standardized direct influence matrix. Two key metrics were extracted: the centrality, which reflects the overall prominence or importance of each factor in the system, and the causality, which indicates whether a variable is mainly a cause (positive value) or a result (negative value) within the network. Here, influence degree represents the degree to which a factor influences others, while affected degree represents the degree to which it is influenced by others. These values allow for deeper insights into the roles and positions of variables in the causal structure, ensuring methodological transparency and replicability.

**Table 10:** DEMATEL causal relationship calculation results.

Comprehensive impact matrix	Impact value	Affected value	Centrality value	Causality value
COG	1.115	0	1.115	1.115
ATT	0.072	0.325	0.397	−0.253
SUB	0.12	0.348	0.468	−0.229
PER	0.154	0.327	0.481	−0.173
INT	0	0.461	0.461	−0.461

First, regarding centrality measures, rural revitalization cognition (1.115) ranks highest, followed by perceived behavioral control (0.481), subjective norm (0.468), participation intention (0.461), and behavioral attitude (0.397). Rural revitalization cognition has a causality degree of 1.115 (>0) and an affected degree of 0, indicating it is a strong causal factor within the system. This underscores its pivotal role in influencing other variables, particularly in shaping behavioral attitude, subjective norm, and perceived behavioral control—consistent with SEM results that highlight cognition as a fundamental driver in forming these constructs.

Higher levels of rural revitalization cognition enhance students’ sensitivity to social expectations and improve the accuracy of their self-assessments (Bao and Ma, 2021). This aligns with the view that increased cognition fosters systematic thinking, enabling students to analyze the background, objectives, and impacts of rural revitalization policies and form a comprehensive cognitive framework. Such understanding strengthens awareness of social norms and confidence in personal abilities (Evans and Stanovich, 2013). Interview feedback supports these findings, with participants in the “Furong Scholars • Rural Revitalization” program reporting that understanding the strategic significance and practical pathways of rural revitalization clarified the alignment between policy goals and personal values, enhancing their initiative and confidence. Students who had participated in policy briefings or rural field visits often described a cognitive “breakthrough moment.” One commented, “Understanding the national strategy made everything click—it wasn’t just volunteer work anymore.” This transformation in understanding functioned as the foundation for attitude change and enhanced sense of control. Moreover, higher cognition levels allow students to better anticipate challenges and devise strategies to address them.

Secondly, the causality degree of participation intention is −0.461 (<0), with an affected degree of 0 and an influence degree of 0.461, indicating that it is a strong outcome variable. According to the Theory of Planned Behavior (TPB), participation intention is a crucial antecedent for predicting actual behavior, as it directly influences individuals’ decisions to act. Higher participation intention generally reflects stronger motivation and more favorable attitudes, increasing the likelihood of translating intention into behavior. Thus, to influence a group’s behavior through external interventions, the primary target should be to enhance participation intention. Only by stimulating this intention can interventions achieve their desired outcomes and facilitate actual behavioral change. From this perspective, changes in participation intention serve as a key indicator for evaluating the effectiveness of intervention measures (El-Zein et al., 2006). Specifically, if participation intention significantly increases after implementing interventions, the likelihood of individuals engaging in the target behavior, either in the short or longer term, rises correspondingly. This logic aligns with TPB, wherein external interventions shape behavioral intentions that subsequently drive action. However, participation intention alone does not guarantee actual behavior. The transition from intention to action is not always seamless, and it is insufficient to assume that increased intention will inevitably lead to immediate behavioral change. Even if interventions enhance intention in the short term, their long-term effectiveness may remain uncertain if they do not cultivate enduring intrinsic motivation. Therefore, it is essential to continuously monitor changes in university students’ participation intention to assess the short-term impacts of interventions and to gather data for evaluating long-term sustainability. Relevant authorities and universities should track both participation intention and actual behavior to determine whether interventions produce lasting and meaningful outcomes.

Third, behavioral attitude, subjective norm, and perceived behavioral control show affected degrees of 0.072, 0.120, and 0.154, respectively; influence degrees of 0.325, 0.348, and 0.327; and causality degrees of −0.253, −0.229, and −0.173. These values indicate that while these factors significantly influence participation intention, they are more result factors within the system, primarily shaped by causal variables like rural revitalization cognition. This pattern, where causality degrees are negative while influence degrees are positive, suggests these variables act as intermediaries—receiving influence from cognition and in turn shaping intention. In forming students’ willingness to participate, these factors directly determine the strength of participation intention, but their development is influenced by cognitive understanding of rural revitalization, as well as broader social and personal factors. Moreover, shifts in these variables are dynamic, reflecting both external influences and individual characteristics such as personal traits and family background. This is corroborated by interview feedback. Many students indicated their attitude or confidence levels changed only after receiving encouragement or assurance from mentors or program organizers. Comments like “My family’s support made all the difference” and “Once I knew I would not be alone, I felt I could handle it” demonstrate how external validation shapes internal evaluations.

Centrality analysis further indicates that subjective norm and perceived behavioral control play more critical roles in driving participation willingness, whereas behavioral attitude exerts a relatively weaker influence. This is consistent with the SEM findings, suggesting that interventions targeting behavioral attitude may be more challenging due to its multidimensional nature and resistance to rapid change. Conversely, strategies focused on subjective norms and perceived behavioral control may be more effective. College students weigh both internal and external motivations when deciding whether to engage in rural revitalization, considering not only emotional and social value but also social norms and the influence of role models. Interview responses confirm this perspective, as participants noted that their decisions were strongly influenced by parents, teachers, and peers. Additionally, for students new to rural revitalization, support and resources provided by program organizers reduced apprehension and enhanced willingness to participate. Targeting these two dimensions aligns well with the TPB framework, further validating its applicability in understanding college students’ participation decisions.

## 4 Discussion

### 4.1 Theoretical contributions

1 New insights and extensions on the mechanism influencing participation intention

Previous studies have predominantly focused on objective factors such as economic development level, government support policies, and employment environment in explaining individuals’ intentions to participate in rural revitalization (Liu, 2021; Chen and Chen, 2023). This study extends the research perspective by centering on high-quality university students as the core behavioral agents, with particular attention to their subjective cognition and psychological factors in shaping participation intentions. Specifically, this study constructs and validates a psychological chain model of “Cognition → Attitude/Subjective Norm/Perceived Behavioral Control → Intention” (COG→ATT/SUB/PER→INT), which explains 89.9% of the variance in participation intention. These findings not only enrich theoretical discussions on the role of subjective factors in rural revitalization research but also provide a direct psychological mechanism explaining how university students’ participation intentions are formed from the perspective of their internal motivations, thereby improving the theoretical framework on participation behavior in rural revitalization.

2 Application and extension of the Theory of Planned Behavior (TPB) in cross-contextual settings

Unlike prior TPB studies that have primarily focused on validation within single behavioral contexts, this study expands TPB on both theoretical and empirical levels. Theoretically, it incorporates “Rural Revitalization Cognition” (COG) into the TPB model as a precursor driving behavioral attitude, subjective norm, and perceived behavioral control. This design not only echoes the theoretical perspective of “Cognition → Intention” proposed by previous research (M Li et al., 2020; Luo et al., 2022) but also enriches the explanatory dimensions and applicability of TPB. Empirically, the expanded TPB model was applied to the cross-context of “university students’ participation in rural revitalization” and systematically tested using structural equation modeling (SEM), mediation analysis, and DEMATEL methods. Results indicate that students’ participation intentions vary by individual and social characteristics, with the COG variable demonstrating the highest centrality index (1.115), and the path effects of SUB and PER being significantly stronger than that of ATT.

These findings not only deepen the micro-level understanding of mechanisms influencing participation intention but also provide specific psychological mechanism supplements and targeted intervention guidance for macro-level practical recommendations proposed by existing studies [e.g., breaking cognitive barriers (Wang et al., 2023); reshaping young elites’ views on rural development (Yan-Xia, 2025)]. Moreover, they call for a more critical re-examination of the relative influence of subjective norm and perceived behavioral control compared to behavioral attitude in collectivist cultural contexts such as China. The stronger predictive power of subjective norm may reflect the significant role of familial expectations, institutional advocacy, and peer influence in youth decision-making processes, aligning with Confucian values that prioritize social harmony and collective conformity. Likewise, the pronounced effect of perceived behavioral control may stem from the pragmatic orientation of Chinese students, who often assess the feasibility, safety, and accessibility of participating in rural programs—particularly given the persistent urban–rural divide. These culturally embedded psychological tendencies highlight that interventions aiming to enhance collective endorsement and perceived competence may be more effective than those focused solely on shaping individual attitudes. Furthermore, by embedding these findings within the broader framework of the Theory of Planned Behavior (TPB), this study extends the application boundaries of TPB to the domain of complex social behaviors and provides robust empirical evidence for designing more nuanced and culturally responsive intervention strategies.

### 4.2 Practical implications

1 Implementing differentiated and precise incentive policies

This study finds that factors such as gender, household registration status, academic background, educational level, and political orientation significantly influence university students’ intentions to participate in rural revitalization. Specifically, female students, those with urban household registration, humanities and social sciences majors, higher educational levels, and stronger political consciousness exhibit higher participation intentions. Therefore, policymakers at all levels should design differentiated and precise incentive policies tailored to diverse student groups. For instance, government departments and universities can provide additional support to male students, those from rural households, lower-grade students, and non-humanities and social sciences majors by establishing special funding programs, offering volunteer service subsidies, and expanding rural internship and research opportunities. In concrete terms, male students and STEM majors could be incentivized through research-based rural innovation competitions, credit-linked practice modules, and exposure to tech-driven rural projects that align with their disciplinary strengths. For lower-grade students, early exposure through immersive summer camps or experiential short-term projects may gradually build up motivation. For students with rural hukou, highlighting role models who have successfully returned to rural hometowns or framing participation as a form of giving back may enhance emotional resonance and social recognition. These differentiated strategies can more precisely address the motivational and capability gaps across groups and enhance the inclusiveness and effectiveness of rural participation initiatives.

2 Enhancing university students’ cognition of rural revitalization

The findings highlight that cognition of rural revitalization is the primary driving factor influencing participation intention. Therefore, it is necessary for national and local governments, together with universities, to establish collaborative mechanisms to enhance students’ understanding of rural revitalization. Governments can utilize mainstream media, online platforms, short videos, and public service campaigns to disseminate rural revitalization concepts and strengthen public, especially youth, recognition. Universities can systematically integrate rural revitalization content into general education, ideological and political courses, and practical teaching, and develop special lectures, elective courses, and credit-based rural practice programs to facilitate comprehensive student understanding of relevant policies and theories, thus stimulating intrinsic motivation to participate. Additionally, aligning cognition-building initiatives with students’ career aspirations can further enhance relevance. For instance, integrating rural case studies into career planning courses, or inviting successful alumni who engaged in rural revitalization to share their pathways, can bridge the gap between rural engagement and long-term personal development goals. This not only strengthens students’ psychological identification with rural revitalization but also alleviates perceived risks of participation.

3 Focusing on synergistic guidance along key driving pathways

The study finds that behavioral attitude, subjective norm, and perceived behavioral control constitute the core pathways affecting participation intention, with subjective norm and perceived behavioral control having particularly significant effects. Therefore, intervention measures should focus on synergistic guidance along these key pathways. Governments and universities can jointly create supportive social environments by mobilizing families, schools, and wider society through policy advocacy, role model promotion, and campus cultural activities to strengthen the influence of subjective norms. In the Chinese context, leveraging the influence of respected social figures (e.g., influential professors, student leaders, or community heroes) to endorse rural participation can amplify normative pressure. Moreover, using digital platforms to share compelling narratives of student involvement may further normalize such behavior among peers. At the same time, simplifying participation processes, clarifying practice pathways, enhancing skills training, and optimizing the matching between projects and majors can reduce practical barriers, enhance students’ perceived behavioral control, and ultimately foster stronger self-efficacy and confidence in their internships and future career development. In particular, creating discipline-matched rural engagement tracks—e.g., design majors working on rural branding, medical students participating in health camps—can reduce mismatch anxiety and enhance perceived control. Providing training certifications, performance recognition, and follow-up career counseling can further consolidate students’ self-efficacy and sense of long-term benefit.

## 5 Conclusion

Drawing on the extended Theory of Planned Behavior (TPB), this study integrates quantitative and qualitative methods to examine the micro-level motivational mechanisms underlying college students’ willingness to participate in rural revitalization. The main conclusions are as follows:

1 Overall trends and individual differences in participation willingness

Individual and social characteristics significantly shape college students’ intention to engage in rural revitalization. Female students, urban residents, humanities and social sciences majors, those with higher educational levels, and individuals with stronger political awareness display higher participation willingness. These insights offer theoretical guidance for designing targeted and precise policy interventions.

2 Central role and systemic influence of rural revitalization cognition

Rural revitalization cognition emerges as a core “strong causal factor” with the highest centrality in the system, exerting significant positive effects on behavioral attitude, subjective norm, perceived behavioral control, and participation intention. Enhancing students’ cognitive understanding of rural revitalization thus represents a crucial entry point for interventions at national, regional, and institutional levels.

3 Key motivational pathways and mediating psychological mechanisms

Behavioral attitude, subjective norm, and perceived behavioral control significantly predict students’ willingness to participate and partially mediate the link between rural revitalization cognition and participation intention. Among these, subjective norm and perceived behavioral control exert stronger effects and explain more variance than behavioral attitude. These findings suggest that combining social support with efforts to strengthen individuals’ perceived capability—thereby enhancing subjective norms and perceived behavioral control—is essential for fostering students’ willingness to engage in rural revitalization.

## Data Availability

The original contributions presented in the study are included in the article/supplementary material, further inquiries can be directed to the corresponding author/s.

## Author’s note

The “Furong Scholars • Rural Revitalization” public welfare program is a major initiative launched in Hunan Province to support national strategies. Jointly organized by the China Youth Development Foundation, the Communist Youth League of Hunan Province, and Hunan China Tobacco Industry Co., Ltd., the program focuses on fostering industries and talent. It encompasses sub-projects in public services and industrial research across education, health, culture, and related fields. By establishing partnerships between universities and designated counties, the program implements targeted interventions, encouraging students to engage in rural revitalization through volunteer activities.
